# Long Island College Hospital

**Published:** 1860-01

**Authors:** 


*Long Island College Hospital.*—It is with pleasure that we announce the complete organization of this institution, with a full corps of Professors, forming a faculty as efficient, perhaps, as any in the country. This has been attempted several times, but has not been before brought to a successful termination. The reasons of these failures are more of a local than a general interest; and it will not be wondered at by any who have been connected with medical schools, that difficulties should have arisen in the way of the establishment of this. Difficulties always do present themselves, and, generally, when least anticipated, in the course of such undertakings; but the final success which has crowned the efforts of those who have interested themselves in this project, at least in the securing of a powerful faculty, would repay even much more time and labor than they have expended. With these matters, however, we have nothing to do. About the former organizations and the reasons of their failure, we know comparatively little, and therefore will only look at the institution in its present aspect. Indeed, we should not have referred to this had our attention not been called to it by late communications in some of the medical journals.

Though schools are often affected by circumstances of location, of prestige, or by things independent of the efficiency of their teaching, yet success cannot be attained without a thorough system of instruction. Rival institutions may accuse each other of bringing other influences to bear upon their classes than those of legitimate competition. No doubt, skillful management, and the like, have much to do with the success of a school; but if it have not the foundation of a powerful corps of teachers, all the management in the world will not insure success. Medical teaching has, however, undergone a marked change within the last few years. One department, Physiology, may be said to have been actually created within fifteen years; and all are now more practically and efficiently taught. In the practical departments, clinical teaching occupies a much more prominent place than it did in old times. Obstetrics, which is sufficiently plain when demonstrated and illustrated, but invested with a mystery sometimes never penetrated when merely talked about, is now taught in the proper way. A corresponding improvement has also taken place in the elementary departments; and there is now no excuse for a graduate if he be not qualified, both by precept and actual experience, to practice his profession intelligently and satisfactorily.

From our knowledge of the gentlemen composing the faculty of the Long-Island school, we are satisfied that the course of instruction will be as complete as possible—both from the eminent qualifications of the teachers, and the facilities for teaching which they have at their command. With half of the faculty, we have had the privilege of an intimate personal acquaintance, and the reputation of the others is a sufficient guaranty of their ability to teach their respective branches. Three members of the faculty are from the city of Brooklyn—Drs. Hutchinson, Chapman, and Enos, who respectively fill the chairs of Surgical Anatomy, Materia Medica and Therapeutics, and Descriptive Anatomy. Dr. Trask, occupying the chair of Obstetrics, is from White Plains, and is well known to the profession, in his department. The other gentlemen are well known to the profession as successful teachers, and have had long experience as lecturers on their respective branches. We cannot but commend the judgment and want of prejudice of the council in securing the best talent in the country, irrespective of any other consideration, in starting the school. Of the qualifications of Prof. Flint, in the chair of Practice, we have nothing to say. He has long been before the medical world as a teacher, having lectured of late years in two of the most flourishing institutions of the South, and as the author of works on Continued Fevers, on Diseases of the Respiratory Organs, and, lately, a treatise on the Heart, besides essays of a less voluminous character. Prof. Hamilton, in the department of Surgery, is also known, both as a lecturer and a writer. His work on Fractures and Dislocations, which is just completed, will add much to the reputation which he has already acquired, as a brilliant lecturer and profound and accomplished writer. In commenting upon these chairs, the most important of all, we can say from actual knowledge, that the teaching in their departments will be *practical* in the highest degree. The Hospital, which is in the same building as the College, affords an abundance of material for clinical illustration, which is the *only* means of effectual teaching. With Prof. Doremus we have had the pleasure of being associated for the past year; and though we are tolerably familiar with the mode of teaching chemistry in medical institutions, we can truly say that we never saw it taught so well as in the New York Medical College. This branch is usually the most uninteresting of all to the mass of students; but we conceive that this is because it is seldom presented to them in a tangible and interesting form. Chemistry should be made a great feature in a medical college; and by the selection of Dr. Doremus for this chair, we see that the Council of this school are determined to make it so. Lastly, Physiology, created almost by late investigators, the most attractive of all branches, and, as experience is daily demonstrating, underlying the whole of rational medicine, is to be filled by Prof. Dalton. We might almost say that Prof. Dalton has originated the teaching of physiology in America; and, as yet, with the humble exception of ourselves, he is the only teacher who attempts to illustrate it by vivisection and actual demonstration. In regard to this branch, we are absolutely certain that the day is not far distant when it will be considered as necessary to demonstrate physiology, and illustrate it by experiment, as it is now indispensable to have a subject before the Professor of Anatomy, or to perform experiments in chemical lectures.

In conclusion, we congratulate the Council of the Long-Island school on the faculty which they have obtained, and we commend their judgment in making a point that *all* the branches shall be made demonstrative and practical. In this, they have a most powerful element of the success which we wish them most heartily.

